# Atomistic structural ensemble refinement reveals non-native structure stabilizes a sub-millisecond folding intermediate of CheY

**DOI:** 10.1038/srep44116

**Published:** 2017-03-08

**Authors:** Jade Shi, R. Paul Nobrega, Christian Schwantes, Sagar V. Kathuria, Osman Bilsel, C. Robert Matthews, T. J. Lane, Vijay S. Pande

**Affiliations:** 1 Department of Chemistry, Stanford University, Stanford, CA, 94305, USA; 2 Adimab, LLC, Lebanon, NH, 03776, USA; 3 Sanofi Genzyme, Cambridge, MA, 02142, USA; 4 Department of Biochemistry and Molecular Pharmacology, University of Massachusetts Medical School, Worcester, MA, 01655, USA; 5 SLAC National Accelerator Laboratory, Menlo Park, CA, 94025, USA.

## Abstract

The dynamics of globular proteins can be described in terms of transitions between a folded native state and less-populated intermediates, or excited states, which can play critical roles in both protein folding and function. Excited states are by definition transient species, and therefore are difficult to characterize using current experimental techniques. Here, we report an atomistic model of the excited state ensemble of a stabilized mutant of an extensively studied flavodoxin fold protein CheY. We employed a hybrid simulation and experimental approach in which an aggregate 42 milliseconds of all-atom molecular dynamics were used as an informative prior for the structure of the excited state ensemble. This prior was then refined against small-angle X-ray scattering (SAXS) data employing an established method (EROS). The most striking feature of the resulting excited state ensemble was an unstructured N-terminus stabilized by non-native contacts in a conformation that is topologically simpler than the native state. Using these results, we then predict incisive single molecule FRET experiments as a means of model validation. This study demonstrates the paradigm of uniting simulation and experiment in a statistical model to study the structure of protein excited states and rationally design validating experiments.

Globular proteins are chains of amino acids that fold into complex, three-dimensional tertiary structures. These structures then fluctuate in a dynamic manner in order to perform a diverse set of functions essential to life. In specific cases, folding is known to occur via transient intermediates, high-energy excited states that are marginally populated before the protein reaches the native state. For example, during the folding of β/α repeat proteins, such as the ubiquitous (β/α) TIM barrel^1^ and the Rossmann fold^2^ motifs, observable kinetic intermediates have been identified. It has been suggested these arise due to the collapse of the unfolded protein into off-pathway excited states on the sub-millisecond timescale that must backtrack before productive folding to the native state can occur^3^. Beyond folding, excited states have been shown to play critical roles in various aspects of protein function including ligand binding and enzyme catalysis^4^,^5^,^6^,^7^,^8^,^9^,^10^,^11^. Detailed structural knowledge of these excited states would therefore greatly advance our general understanding of protein dynamics.

Characterizing excited states poses significant challenges. Due to their rapid formation, transient nature, and multiplicity of populations, excited states are difficult to interrogate experimentally at high resolution. For example, the folding intermediate of the model β/α-repeat protein CheY has been identified using techniques such as circular dichroism (CD) and small-angle X-ray scattering (SAXS)^3^, but atomic-resolution structural details are still unavailable. Due to the lack of high-resolution data, experiments can only impose weak structural constraints on the excited state. For example, two structures that differ considerably at atomic resolution may have similar overall shape and size and be equally consistent with an experimental SAXS profile, making it impossible to decide, based on experiment alone, which should correspond to the true excited state. Also, the excited states of proteins may be structurally heterogeneous and exhibit rapid fluctuations on nano- to microsecond time scales, rendering a static description incomplete. In this case, an excited state structural model must capture an ensemble of structures^12^.

In the regime where a structural ensemble is experimentally under-determined, it is not possible to directly deduce a unique ensemble from the experimental data. Instead, a common alternative is to simulate an ensemble of structures that is consistent with experiment to use as an inference of the true ensemble^13^,^14^. This involves either using a biased sampling algorithm in which the experimental data are used as constraints to steer a simulation towards “important” conformations^15^,^16^,^17^,^18^,^19^, or refining a randomly sampled set of structures by picking out those that are most consistent with experiment^20^,^21^,^22^,^23^,^24^. The success of either of these methods strongly depends on the quality of the available experimental data. In the data-rich regime, in which the experimental data can provide strong structural constraints on the protein, the inferred ensembles will likely be reasonably “close” to the true ensemble. On the other hand, if one is instead in the data-poor regime, where the experimental data (e.g. CD, SAXS) imposes weak constraints on structure and the ensemble is highly under-determined, there is an inherent danger of sampling an ensemble consistent with experiment but structurally distant from the true ensemble. Under these circumstances, one method to improve the accuracy of prediction is to incorporate strong prior information to distinguish between degenerate ensembles.

Here, we present an approach for atomistic excited state structural inference in the data-poor regime that uses all-atom molecular dynamics (MD) simulation data as a prior for the excited state ensemble, which is then subsequently refined against experimental data. We first extensively simulate the system using all-atom MD with the distributed computing platform Folding@home. This data is then used to construct a kinetic model of the protein dynamics, a Markov State Model (MSM), which allows prediction of the excited state ensemble at the experimentally determined timescale. This approach enables us to obtain a time-resolved representative ensemble of structures, with each structure weighted according to the underlying dynamics modeled by the force field. Our approach has some similarities to previous studies in which MD is used to generate ensembles of structures that were then refined against experimental results^25^,^26^,^27^. However, in contrast to these methods, which used coarse-grained and/or biased MD methods to improve sampling, the massive computing power afforded by Folding@home allows us to simulate the system using an all-atom, unbiased representation to timescales orders of magnitude beyond which the excited state is experimentally observed. This allows us to take advantage of the strong kinetic prior information in our simulation model to generate not only a candidate set of structures, but also make a robust statistical prediction about the relative importance (population) of each structure in the excited state ensemble, which we can then refine against the experimental data. Furthermore, using the structural insight obtained from our atomistic model, we are able to straightforwardly design incisive experiments to cross-validate our predictions, which would otherwise be very difficult due to the lack of high-resolution experimental information about the excited state.

The system we studied is CheY, a β/α repeat model protein responsible for facilitating chemotaxis in *Escherichia coli*^28^. Its native state consists of a central all-parallel β-sheet of five strands surrounded by five α-helices. The sheet is non-sequential, with a strand ordering of β2-1-3-4-5, with the N-terminal strand β1 situated in between β2 and β3. The packing of the helices against the central sheet are stabilized by two buried hydrophobic clusters^3^. Experimentally, it is known that during refolding, CheY collapses on the ~100 μs timescale into an off-pathway “burst-phase” intermediate that has been identified by far-UV CD and fluorescence spectroscopy but has not yet been characterized at high resolution^3^,^29^. This species has measurable stability and retains ~95% of the native CD intensity at 222 nm and 80% of the native fluorescence intensity after 5 ms of refolding. Small differences in the far-UV burst phase CD spectrum between the intermediate and native states were attributed to the perturbation of an exciton coupling between aromatic side chains, indicating that a quartet of phenylalanine residues F8, F30, F53, and F124 are packed loosely or irregularly with respect to the native state^3^. A study by Hills and Brooks in the folding of CheY was simulated under a native-centric Gō-type potential also showed evidence of early kinetic frustration of CheY into a species with a prematurely formed interface between the N and C-terminal halves of the protein (α2-3 and β3-4) and relatively unstructured C-terminus^30^. These combined experimental and simulation results suggest an excited state with a high degree of native secondary structure, but a loosely packed core. However, a definitive high-resolution structure remains elusive.

Our approach to inferring structural features of the excited state of CheY at atomic detail involves first extensively simulating the protein using MD, and then using the data to build a Markov State Model (MSM) of the dynamics, which partitions the sampled protein conformations into discrete “microstates” based on kinetic criterion and computes transition rates between them. Using this model, we can infer the CheY conformational ensemble at the timescale in which the excited state is observed experimentally (5 ms) to generate a prior for the excited state ensemble. We then refine our model by simulating an average experimental observable for the prior, and then repeatedly perturbing the ensemble weights to improve agreement between this prediction and experimental data. This method of excited state inference is attractive because one can take advantage of the powerful prior information encoded in the MD force field to generate atomistic models of non-equilibrium ensembles even when the resolution of the experimental data is limited. In addition, an ensemble model gives a more realistic description of the excited state, which may be a highly heterogeneous ensemble of protein conformations.

Our excited state model contains significant non-native contacts between strands β2 and β3. This structure is topologically simpler than the native β2-β1-β3 arrangement and therefore provides a possible explanation for the presence of an intermediate in the folding of CheY. SAXS experiments do not guarantee the presence of this feature – it is a result of combining information from SAXS with the MD force field – so in order to validate this model we propose a FRET experiment based on our results that could directly confirm our prediction.

## Results

### Molecular dynamics and subsequent ensemble refinement provide an inference of the excited state at atomic detail

We inferred the excited state ensemble of a stabilized variant of CheY, F14N^29^, herein referred to as CheY*, as follows (also see Methods). First, we constructed a MSM, a kinetic model of the protein dynamics, from the raw simulation data (see Methods). Through this process, we obtain a set of representative conformations sampled by the simulation, or “microstates”, and a model for their populations as a function of time.

For each structure, we then simulate its SAXS profile using the predictor CRYSOL^31^. This choice was made as a result of a benchmarking study we performed that compared the performance of many different SAXS predictors in simulating the native state CheY SAXS profile, with CRYSOL giving the best agreement (see Methods and SI Fig. 1).

Starting from a prior of equal populations in each microstate, we then refine an ensemble of microstates against experimental SAXS data of the 8M urea denatured state of CheY* using an established literature procedure known as EROS^32^. From this procedure, we obtain a reweighted ensemble of states that serves as an estimate of the unfolded state ensemble, which we denote **E**_**U**_ (SI Fig. 5). Then, we used the MSM to propagate **E**_**U**_ forward in time to estimate the microstate populations after 5 ms of simulated refolding. These dynamics are meant to model the double jump refolding experiment performed previously in which CheY* begins in a high-denaturant environment (8M urea) and then is rapidly diluted to folding conditions and refolding under folding conditions^3^, SI Fig. 7b. This resulting ensemble, which we denote **E**_**MSM**_, serves as the prior for the experimentally observed excited state. Finally, we refined **E**_**MSM**_ against the experimental SAXS profile after 5 ms of refolding to obtain the ensemble incorporating both experimental and prior information, which we denote **E**_**SAXS**_. To maximize the accuracy of our MD simulations, we generated aggregate simulation data for CheY* (42 ms) many times longer than the timescale of collapse to the excited state (100 μs) to thoroughly sample the excited state conformational space. In addition, the force field, solvent model, and simulation temperature used for the CheY* simulations were also benchmarked previously in separate studies^33^,^34^. We also confirmed that the amount of data collected was sufficient for a converged dynamical model (SI, Fig. 8).

To test the accuracy of the simulation kinetics against experiment, we compared the MSM refolding process from **E**_**U**_ to **E**_**MSM**_with a time resolved SAXS refolding experiment (see Methods). The MSM predicts the protein transitioning from an expanded unfolded state (Rg = 37.3 Å) to a compact state with Rg = 21.9 Å by 200 μs that is stable for at least 10 ms, but still significantly more expanded than the native state (Rg = 15.3 Å). These predictions are consistent with experiment, in which the protein was observed to collapse from the unfolded state (Rg = 35.5 ± 1.5 Å) rapidly to a “burst-phase” excited state with Rg = 25.3 ± 2.2 Å within 142 μs and further compact to a steady state with Rg = 22.6 ± 2.0 Å by 1 ms that is stable for at least 18 ms^35^. Thus, we concluded that **E**_**MSM**_was a reasonable initial guess of the experimentally observed excited state.

**Figure 1:**
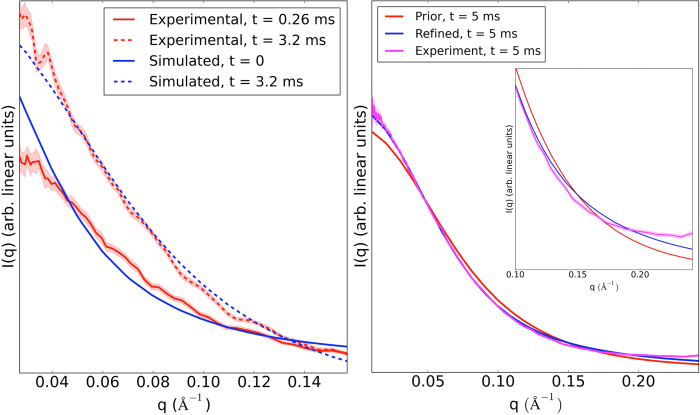
Raw MD simulations agree with time-resolved SAXS data, refinement of MD simulations with the experiment results in an improved model. (*Left*) The predicted time evolution of the ensemble SAXS profile by the MSM (blue) was compared to a continuous-flow SAXS experiment (red) as a way to benchmark the predicted excited state prior **E**_**MSM**_. Due to the dead time of the instrument, there was no t = 0 time point, and only changes from t = 0.26 ms onward could be observed. The simulated profiles at t = 0 and t = 3.2 ms were rescaled independently to the experimental t = 0.26 ms and t = 3.2 ms profiles respectively. The Rg of the experiment and simulation at the millisecond timescale agree within error (22.7 ± 2 Å vs. 21.9 Å respectively). (*Right*) Subsequent refinement of **E**_**MSM**_ using the EROS algorithm gives a new ensemble **E**_**SAXS**_, that shows improved agreement with experiment. The experimental standard deviation due to repeat measurements is represented as the shaded area around the experimental SAXS profile.

**Figure 2:**
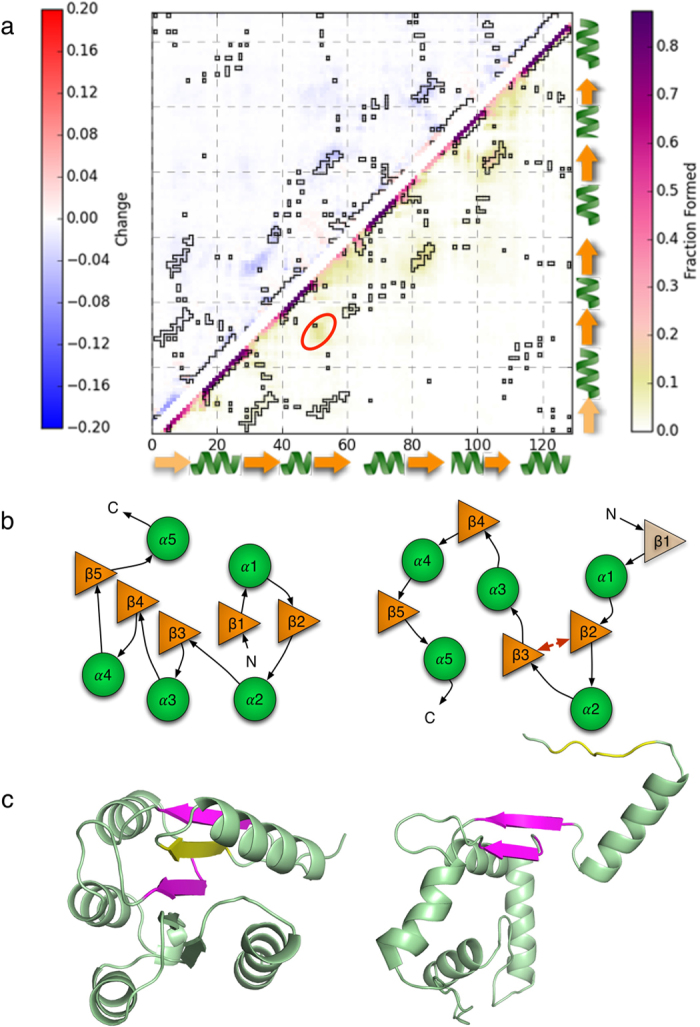
The combined MD and SAXS excited state ensemble exhibits non-native contacts and an unstructured N-terminus. (**a**) The contact map for the refined excited state **E**_**SAXS**_, with native contacts outlined in black, is shown in the bottom right. The top left is the difference map **E**_**SAXS**_ - **E**_**MSM**_, showing the effect of introducing experimental information in the ensemble. The most notable features of the excited state are the presence of non-native contacts between β2 and β3, populated to about 15% in **E**_**SAXS**_ (circled in red), and the almost complete absence of β1 from the central sheet, as indicated by the very small population (<5%) of β1-2 and β1-3 contacts. These features indicate the excited state structure is stabilized by non-native contacts and exhibits a loose β1/α1 N-terminal tail. (**b**) Illustration of the loose β1/α1 tail stabilized by non-native β2-3 contacts, leading to an unstructured N-terminus (right), compared to the native state, in which β1 is situated in between β2 and β3 (left). The interface and C-terminus are also mostly unstructured, but more are still significantly more structured (20%) relative to the N-terminus (<5%). (**c**) Representative native and excited state structures pulled from the structural ensemble. The excited state structure shown (right) is the one with the highest population in **E**_**SAXS**_ that possessed non-native β2-3 contacts. The loose β1/α1 tail is stabilized by β2-3 contacts (magenta) that exclude β1 (yellow) from the central sheet. The interface and C-terminus are also unstructured, as seen from the absence of the β3-5 portion of the central sheet.

After refining **E**_**MSM**_, the resulting ensemble **E**_**SAXS**_ gives a SAXS profile that has significantly better overall agreement with the 5-ms refolded experimental SAXS data (Fig. 1, right). The slight deviations from the experimental profile for q > 0.17 is due to the regularization penalty imposed by the EROS refinement algorithm that restricts level of refinement in order to prevent overfitting to the experimental data (see Methods). Significantly better agreement in this region is achievable by eliminating this penalty (SI, Fig. 2), but due to the prediction error likely incurred by using default CRYSOL parameters (seen explicitly for the native state in SI, Fig. 1), it is likely that such a solution results from overfitting the experimental data with our ensemble. Therefore, we decided to select an intermediate value of the regularization penalty (see Methods) that allows for reasonable refinement but also restrains the ensemble to be relatively close to our prior **E**_**MSM**_.

### CheY*’s excited state has an unstructured N-terminus stabilized by non-native contacts

The excited state model of CheY* can be represented by the ensemble contact map of the refined ensemble, **E**_**SAXS,**_and the difference map between **E**_**MSM**_ and the prior **E**_**MSM**_, which shows the effects of the refinement procedure. Both of these contact maps are shown in Fig. 2. Our inferred prior for the excited state, **E**_**MSM**_, is mostly unstructured, exhibiting few long-range contacts. Both the interfacial β3-4 contacts and C-terminal β4-5 contacts are formed in 16% and 22% of the ensemble respectively, which is similar to the degree of structure in the folding intermediate observed by Hills and Brooks in these regions (~20%)^30^. In addition, the native helices, while present in >90% of the ensemble, are packed more loosely in the excited state when compared to the native state, with less than 5% of the ensemble showing fully natively packed α2/α3, α3/α4, or α1/α5 interfaces. The C-terminal helix α5 is also rarely in van der Waals contact with the central sheet, with <10% of the ensemble having native hydrophobic contacts between β3-5 and α5.

Notably, the least natively structured region of **E**_**MSM**_ is the N-terminus. Less than 5% of the total population has β1 located between β2 and β3 in the native β2-1-3 topology. Furthermore, a significant fraction of the ensemble (16%) has a non-native two-stranded parallel sheet formed between β2 and β3 that directly inhibits β1 from assuming its native position. β1 likewise shows significant non-native helical propensity (~50%), and native α1/β2 and α1/β3 packing are both less than 10%. These features are indicative of an unstructured, non-native N-terminus with a loose β1/α1 tail.

Refinement of **E**_**MSM**_to **E**_**SAXS**_ slightly decreases the overall contact density, indicating that incorporation of the experimental data serves to reduce the ensemble’s compactness and structure. We speculate that this result is due to the experiment attempting to correct the tendency of implicit solvent models to over-stabilize compact protein conformations^34^. However, the aforementioned structural features of **E**_**MSM**_are qualitatively well preserved in **E**_**SAXS**_. The β2-3 contact density only slightly decreases to 12%, the native N-terminal β2-1-3 topology remains almost completely absent, and the interfacial and C-terminal central sheet contacts are still only marginally formed. To address the possibility that the similarity between **E**_**SAXS**_and **E**_**MSM**_ may be due to insufficient refinement against the SAXS experiments, we performed an alternative refinement using no regularization penalty beginning from an intentionally bad choice of prior, the unfolded ensemble **E**_**U**_, which contained only minimal amounts of these structural features. This refinement gives an ensemble similar to **E**_**SAXS**_ with unstructured N-terminus and marginally formed interface, and notably upweights the non-native β2-3 contact density from 4% to 16% (SI, Fig. 6). This suggests that the structural features seen in **E**_**SAXS**_ and **E**_**MSM**_, *i.e.* an unstructured N-terminus with non-native β2-3 contacts and loose β1/α1 tail, and marginally formed interface and C-terminus, are reinforced by the experimental results and do not arise exclusively due to the prior.

**Figure 3:**
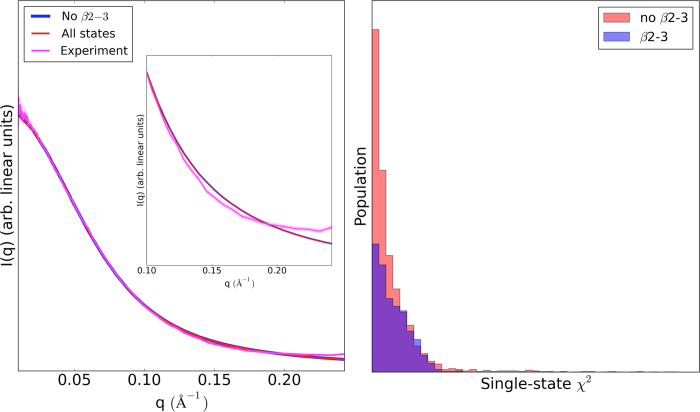
β2-3 contacts are not necessary to describe the SAXS experiment. (*Left*) Two separate refinements from the unfolded prior **E**_**U**_, with and without β2-3 states, were performed and the resulting SAXS profiles were compared to experiment. The refined ensembles are identical within experimental error, indicating β2-3 states are not required in the ensemble to give consistency with experiment. The experimental standard deviation due to repeat measurements is represented as the shaded area around the experimental SAXS profile. (*Right)* A histogram of mean-squared errors between the simulated SAXS profile of each CheY* MSM microstate from the experimental 5-ms SAXS profile indicates that states with the β2-3 feature (blue) are not necessarily in better agreement with experiment compared to states without the feature (red).

Finally, to test how much our choice of regularization penalty affects our prediction of the excited state, we computed the ensemble contact maps of excited state ensembles resulting from refinements using varying strengths of the regularization penalty. We observed that the contact maps are qualitatively similar, and the non-native β2-3 contact density in general remains approximately constant with respect to regularization strength (SI, Fig. 3), which suggests that the key structural features of the excited states are mostly insensitive to degree of regularization. The exception to this trend is when regularization is completely removed, at which point β2-3 contacts sharply increases from 12% to 19%. We hypothesize that this represents an over-estimation of β2-3 content as a result of overfitting the experiment.

### Non-native contacts are not necessary to explain experimental SAXS results

Although states with the β2-3 contacts were significantly up-weighted during refinement of the uninformative unfolded state prior **E**_**U**_, it is possible that this feature by itself may not be a necessary component of the ensemble to give good agreement with experiment. To investigate this, we reduced the set of states, removing all states containing β2-3 contacts from **E**_**U**_, and then re-refined the ensemble with EROS. Interestingly, it was found that even in the absence of these states, EROS was still able to reweight **E**_**U**_into an ensemble equally consistent with experiment within error (Fig. 3, left). In addition, we observed that the presence of β2-3 contacts in a member state of each ensemble is seemingly uncorrelated with the agreement of that state’s SAXS profile to experiment (Fig. 3, right). These results indicate that the available SAXS data is of insufficient spatial resolution to specifically resolve the β2-3 feature and is instead monitoring coarser elements of CheY* structure such as perhaps compactness at the N-terminus.

We conclude that currently available experimental data cannot describe the excited state at high resolution. However, by employing an informative prior – the MD force field – we can infer the existence of detailed features such as the presence of non-native β2-3 contacts normally invisible to experiment. By systematically combining information from both MD and experiment, we are able to draw quantitative conclusions about the structure and dynamics of CheY*. In the following section, we predict a single-molecule FRET experiment that could incisively verify or deny the major structural features of our proposed excited state structural ensemble.

### FRET can be used to experimentally cross-validate E_SAXS_

The most notable feature of the excited state ensemble, **E**_**SAXS,**_ is the unstructured N-terminus, with loose β1/α1 tail and non-native β2-3 contacts. We suggest that these features can be positively confirmed by attaching FRET probes to the protein that report on contact between β1, β2, and β3. We simulated two single molecule FRET experiments at two different donor positions, K7 (β1) and K26 (β2), with a common acceptor position M63 (β3) (for simulation protocol, see Methods) and found this set of experiments would be able to distinguish the unstructured N-terminus from the native N-terminus.

**Figure 4:**
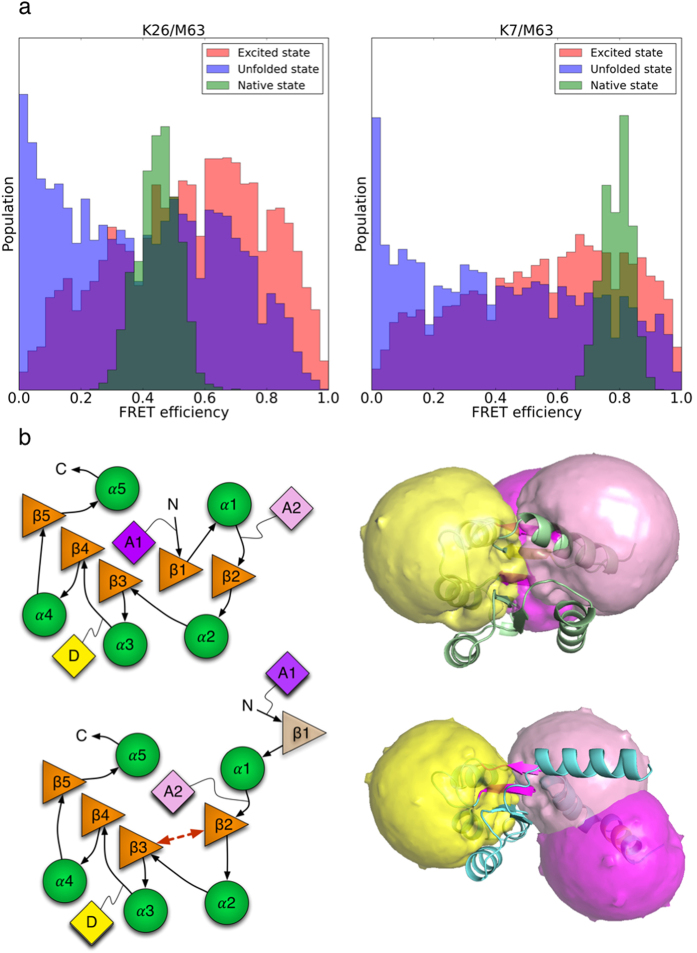
A FRET experiment could validate the proposed excited state structural ensemble. (**a**) Simulated FRET efficiency distributions for the K26/M63 and K7/M63 FRET pairs. Dyes attached to K26 (β2) and M63 (β3) with R_0_ = 38 Å give similar mean FRET efficiencies for the native (0.45 ± 0.06) and excited states (0.55 ± 0.24), indicating that on average, the distance between β2 and β3 is larger by 2.8 Å in the native state This suggests that in the excited state, the β2-α2-β3 portion of the N-terminus is slightly more compact than that of the native state. In contrast, dyes attached to K7 (β1) and M63 (β3) with R_0_ = 42 Å give a significantly higher mean FRET efficiency for the native state (0.79 ± 0.04) compared to the excited state (0.56 ± 0.25), or an approximately 8 Å increase in distance between β1 and β3 in the excited state, suggesting that β1 is loose and not part of the native N-terminal sheet in the excited state. (**b)** *Left:* Cartoon representation of the native (top) and excited (bottom) state features, highlighting the excited state’s non-native β2-3 contacts at the N-terminus in contrast to the native β2-1-3 and relative mean dye positions of K7 (purple, A1), K26 (pink, A2), and M63 (yellow, D). *Right:* Simulated position distributions for the K7 (purple), K26 (pink), and M63 (yellow) dyes for the native state (top), and a conformation containing the non-native β2-3 feature (bottom). Based on our simulations, we predict these two pairs will be effective at inferring the existence of the non-native N-terminal motif of a β2-3 sheet and loose β1 tail. The excited state K26/M63 FRET efficiency will be similar to that of the native state due to similar dye separation distances in both the excited and native states. On the other hand, the excited state K7/M63 FRET efficiency will be noticeably smaller than that of the native state due to the absence of β1 from the central sheet, resulting in a much larger mean distance between K7 and M63.

Our simulations predict the excited and native state K26/M63 FRET efficiencies to be 0.55 ± 0.24 and 0.45 ± 0.06 respectively (Fig. 4a, left). This corresponds to only a small difference in mean β2-3 inter-strand distance, 2.8 Å, suggesting the β2-α2-β3 portion of the N-terminus has a similar degree of compactness in both the native and excited states. On the other hand, the K7/M63 excited state FRET efficiency was predicted to be 0.56 ± 0.25, which was considerably less than that of the native state, 0.79 ± 0.04. This corresponds to a difference in K7-M63 distance of approximately 8 Å, indicating that β1 is unstructured and absent from the central sheet in the excited state. The similarity between the native and excited state-K26/M63 FRET efficiencies coupled with the large difference in the K7/M63 FRET efficiencies can be used to infer a compact excited state N-terminus with unstructured β1. These characteristics are illustrated in plots of the simulated allowed position distributions of the dyes (Fig. 4b). Agreement between future FRET experiments and the results presented here would therefore lend significant support to our model.

To match the dye parameters used in our simulations, a feasible experiment could employ dyes from ATTO-Tec such as ATTO 390/650 (R_0_ = 40 Å)^36^ attached to the protein with a (CH_2_)_5_ linker (length = 15 Å). The experiment must also be able to resolve protein dynamics on at least the millisecond timescale, the timescale during which the CheY* excited state had previously been observed experimentally.

## Discussion

The non-native features in the experimentally refined ensemble, **E**_**SAXS,**_ are consistent with previous observations reported in the literature. An unstructured N-terminus with the loose β1/α1 tail stabilized by the non-native β2-3 sheet, which was the most striking feature of the excited state prediction, is consistent with the hypothesis in literature of a loosely packed quartet of phenylalanine residues forming in the core of the excited state^3^, since F8 and F30 are located on β1 and α1 respectively. Also, in contrast to the native state, in which the K109/P110 prolyl bond is locked in the *cis* isomer due to the hydrophobic packing of α5 against the central sheet, the relatively unstructured nature of the C-terminus of **E**_**SAXS**,_ with very little native packing of α5 against the central sheet, results in the β4-α5 loop being much more flexible. This allows the prolyl bond to isomerize at a rate similar to that of the unfolded state, which is consistent with the experimental observation that the isomerization was approximately urea-independent^3^.

Our results also provide an explanation for the experimentally observed tendency of CheY* to rapidly collapse into a structured intermediate upon refolding, instead of directly reaching the native state. The key observation is that the topological complexity of the excited state N-terminus is noticeably less than that of the native state^37^. It is reasonable to expect sequence-adjacent β2-3 non-native contacts will preferentially form at the N-terminus during the early stages of folding instead of the native β2-1-3 contacts. The propensity of the adjacent α2 and α3 helices to rapidly collapse to form a hydrophobic cluster^35^, could further stabilize this non-native sheet. Partial unfolding, or “backtracking” from the β2-3 sheet and α2/α3 packing would be needed in order for the protein to subsequently reach the native state. This is consistent with the observation of Hills and Brooks that interfacial contacts between α2/α3 had to break before the protein could escape the folding intermediate and continue folding^30^.

While topologically simpler, we posit that the excited state is less stable than the native state due to the fact that the native β2-1-3 arrangement places the hydrophobic strands β1 and β3 in close proximity, allowing burial of those hydrophobic residues, whereas the non-native β2-3 sheet prevents this. Thus, at long timescales, the protein equilibrates and preferentially forms the native β2-1-3 sheet.

In this study we have demonstrated the utility of combining the information contained in empirical MD force fields and experiments limited in resolution. Employing this strategy, we can gain atomistic insight into the structural features of the excited state of CheY*, specifically how a folding intermediate might arise as a result of its topological simplicity relative to the native state. Using the structural information obtained from our model, we then design a specific set of experiments to cross-validate our predictions. We stress that the procedure presented in this study is general and can be used to study ensembles of any protein as long as certain conditions are met: 1) simulation data exists that extensively samples the configuration space of interest 2) experimental data exists at the time scales of interest, and 3) a method exists for accurately predicting the relevant experimental observable from the simulation data. In addition, we strongly recommend that the results obtained from such an approach be used to design and execute an incisive experiment for cross validation purposes. Given that there is growing literature evidence that excited states are ubiquitous in protein folding and also play key roles in various aspects of protein function, the ability to infer information on excited states at atomic detail using this approach could potentially lead to significant progress in understanding protein dynamics as a whole.

## Methods

### Molecular dynamics simulation

A stable mutant of CheY (F14N) named CheY*, was used for this study. The wild-type protein was not used due to its propensity to aggregate, making SAXS experiments impossible. The native state reference structure used for this study was that of wild-type CheY (PDB ID: 3CHY)^38^, to which a single-point mutation was then performed *in silico* to obtain the CheY* sequence. All-atom molecular dynamics of CheY* was performed with GPU-accelerated GROMACS^39^ on the Folding@home distributed computing platform. The AMBERff96 force field^40^ and GBSA-OBC implicit solvent model^41^ were used. Electrostatic and van der Waals cutoffs were both set to 15 Å. The choice of force field and solvent model was based on a previous study, which demonstrated that out of a series of AMBER force fields and implicit solvent models, AMBERff96/GBSA-OBC was able to most consistently reproduce native secondary structure for a set of small peptides^33^. Our system was propagated using the Langevin integrator at 370 K using a time step of 2.5 fs. The simulation was run at artificially high temperature to offset the known tendency of the GBSA implicit solvent model to over-stabilize compact states and give artificially high large free energies of unfolding^34^, Supplementary Information. This combination of AMBERff96/GBSA-OBC/370K has been successful in folding several proteins with very different native state topologies, including NTL9^42^ and ACBP^43^, suggesting that it is not strongly biased towards α-helical or β-sheet structure, and therefore potentially suitable for simulating CheY*, a β/α-repeat protein that contains equal amounts of helix and sheet secondary structure. In addition, we observed that our simulation was able to predict the experimental Rg at 5-ms to within experimental error (see Results). Finally, we observed within our dataset a single folding event in which CheY* transitioned from a partially folded structure to its native state. These observations provide additional evidence that our choice of simulation conditions is suitable for simulating CheY* folding dynamics.

The production simulations were seeded from a total of 3024 starting CheY* conformations ranging from native to unfolded. The K109/P110 prolyl bond was in the *cis* conformation in 1542 structures and *trans* in 1482 structures. Ten independent trajectories with randomized initialized velocities were started from each structure. The final *cis* dataset consisted of 4515 independent trajectories, with lengths ranging from 100 ns to 33 μs. The *trans* dataset consisted of 3815 trajectories, with lengths ranging from 100 ns to 26 μs. The total aggregate dataset was 42 ms.

Because we use the simulation results as a prior to predict the CheY* excited state, the error arising from the simulation, which primarily consists of errors introduced by our choice of simulation parameters (i.e. force field, solvent model, simulation temperature) and parameters chosen during MSM construction, play an important role in determining the uncertainty of our excited state inference. Unfortunately, quantifying the simulation error is not straightforward. We cannot, for example, use the difference between the simulated and experimental SAXS profiles to directly estimate the simulation error. Given a “perfect” SAXS predictor, the simulation error would simply be the difference between the simulated and experimental profiles. However, if errors exist in the SAXS prediction algorithm as well, the simulation error cannot be decoupled and estimated separately.

A quantity that we *can* use to assess the simulation's performance on a coarser level is the simulated radius of gyration (Rg), as this does not depend on an intermediary predictor. The simulated Rg was compared to the Rg extracted from a CF-SAXS experiment (Fig. 1). It was seen that the simulated value at 3.2 ms (21.9 Å) agreed with experiment within experimental error (22.6 ± 2.0 Å). We can use these experimental error bars to set bounds on the simulation error with regards to predicting Rg at the millisecond timescale: [21.9 −1.3 Å, 21.9 + 2.7 Å]. Loosely, this means we can set bounds on how compact (21.9 −1.3 Å) or expanded (21.9 + 2.7 Å) our simulation model predicts the excited state will be. However, due to the fact that Rg encodes less structural information than the SAXS profile, we cannot directly map uncertainty in Rg to uncertainty in SAXS. The best that can be done is to translate these uncertainties in Rg to uncertainty in the slope of the Guinier region of the SAXS profile, (which is used experimentally to compute the Rg).

### MSM construction

A comprehensive review of the theory of MSMs can be found in other literature^44^,^45^. All MSM construction for this study was done using MSMBuilder^46^. The datasets used to build the MSMs were generated by sampling the simulation data at an interval of 1 ns. Clustering of the data was done by first using time-structure independent component analysis (tICA) to identify the slowest degrees of freedom in the data^47^. Then, the data were clustered along these degrees of freedom using the k-centers algorithm and the set of all residue-residue pairwise distances as the metric.

A complication of the MSM construction process for CheY is that its conformational space comprises two slowly interconverting isomeric regimes, defined by the isomerization state of the K109/P110 proyl bond. The isomerization of this bond serves as the rate-limiting step of folding and occurs on a very long timescale on the order of 100 s and is therefore too slow to sample sufficiently by pure simulation. Because this process is several orders of magnitude slower than the rate of collapse from the unfolded to the excited state, we expected its effect in determining the excited state ensemble to be miniscule. Nonetheless, in order to create a comprehensive kinetic model of CheY comprising both isomeric regimes, the CheY* dataset prior to clustering was separated into two subsets, one that included only *cis* conformations of the protein and the other that included only *trans* conformations, and a microstate MSM for each subset of data was created. The *cis* transition matrix had dimensions *n* × *n* and the *trans* transition matrix had dimensions *m* × *m*, where *n* and *m* are the number of microstates for the *cis* and *trans* models respectively. The transition matrices for the *trans* and *cis* regimes were then combined to form a single (*n* + *m*) × (*n* + *m*) block-diagonal transition matrix. This matrix still neglects prolyl bond isomerization. To model the transitions between the two regimes, an empirical transition probability based on an experimental rate constant^3^ was added as off-diagonal-block elements. The rate constant was used to estimate a transition probability using the relationship:


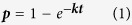


In the above equation, ***p*** corresponds to the transition probability, ***k*** is the experimental rate constant, and ***t*** is the lagtime of the MSM. To determine which pairs of *trans* and *cis* microstates would interconvert, each *trans* microstate was first paired with its closest *cis* microstate as measured by RMSD. Then, for each pair, the *trans-cis* RMSD between the paired states was compared to the mean RMSD between the *trans* and *cis* states and their direct connections in their respective transition matrices. If the *trans-cis* RMSD was less than both *trans-trans* and *cis-cis* RMSDs, it was kept in the model. This serves to eliminate transitions between states with very large RMSD given the lagtime and keep the connections kinetically realistic. The combined *cis/trans* MSM built using this method is meant to directly model the experimental dynamics, and the refolding simulation done to estimate **E**_**MSM**_is meant to correspond exactly to the double-jump refolding experiment on CheY* discussed in a previous study^3^, SI Fig. 7b.

### Steps to inferring an atomistic excited state ensemble

**Figure 5:**
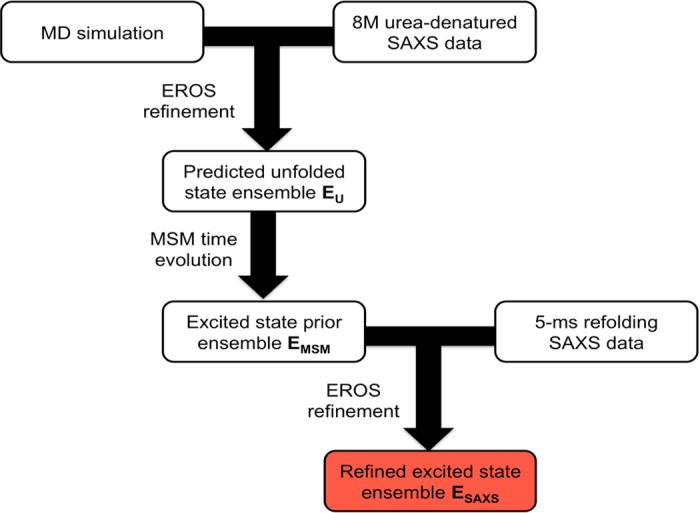
An atomistic inference of the excited state ensemble of CheY* can be made by combining simulation and experiment. (1) An atomic-level inference for the CheY* unfolded state ensemble, **E**_**U**_ is generated by refining the MD data against SAXS data of the 8M-urea-denatured protein. (2) **E**_**U**_ is propagated forward in time according to the MSM for 5 milliseconds, the timescale at which the excited state is experimentally observed to give the excited state prior, **E**_**MSM**_. (3) **E**_**MSM**_ is refined against SAXS data at the 5-ms time point to obtain **E**_**SAXS**_.

The process of ultimately inferring the excited state ensemble **E**_**SAXS**_ consists of the following steps (also see Fig. 5). First, the software package CRYSOL^31^ was used to generate theoretical SAXS scattering intensity profiles for each MSM microstate. CRYSOL was chosen to be the predictor for this study based on a survey we conducted comparing several different SAXS predictors and their ability to reproduce the experimental CheY* native state scattering profile. The predictors tested were CRYSOL, FoXS^48^, AXES^49^, and AQUASAXS^50^. All adjustable parameters for each predictor were set to their default values for the comparison. Overall, CRYSOL gave the best agreement with experiment (SI Fig. 1) and was therefore chosen as the predictor to be used in this study. Individual SAXS profiles for each state in our ensemble were then simulated using CRYSOL with default parameters.

Alternatively, significantly better agreement can be achieved for the native state by using CRYSOL's “fitting mode” with the experimental native state profile as a reference. Using default parameters, we obtain an error of χ^2^ = 13.4 (SI Fig. 1a) between simulated and experimental native state profiles. In contrast, using “fitting mode” and fitting explicitly to the experimental native state profile, we can produce a fit significantly more consistent with experiment (χ^2^ = 1.2, SI Fig. 1b). However, we decided against using these parameters to predict SAXS profiles for our structural ensemble, as previous work has suggested that optimizing CRYSOL’s fit to a single structure and then using these fitted parameters to predict the profiles of other structures can result in overfitting artifacts^48^. We therefore believed it would not be justified to assume the native state prediction error is generalizable to the remainder of our ensemble.

Nevertheless, to address the possibility that using native state-fitted parameters may perform better overall on the ensemble level even taking into account potential overfitting, we computed the perturbation to the predicted SAXS profile of **E**_**SAXS**_ using fitted parameters. To do this, we used CRYSOL to re-compute the SAXS profiles for all structures in our ensemble using the native-fitted parameters, and then computed a new ensemble SAXS profile while keeping the original refined **E**_**SAXS**_ weights constant. This results in only small deviations with **E**_**SAXS**_ that are comparable to the experimental errors (SI Fig. 7). Furthermore, we performed a separate refinement using the fitted parameters, and the resulting ensemble contact map is virtually identical to the **E**_**SAXS**_contact map (SI, Fig. 7). Taking the difference between the two contact maps reveals that the contact density per contact is perturbed by a maximum of 1% (SI Fig. 7). This indicates that the characterization of the excited state is essentially unaffected by which set of parameters we use.

After deciding which parameters to use for CRYSOL, all microstates in the MSM were assigned uniform weights, and a weighted average of all the predicted microstate SAXS profiles was refined against experimental SAXS data of the 8M urea-denatured state of CheY* using the EROS algorithm^32^ to obtain an estimate of the unfolded state ensemble, **E**_**U**_. This ensemble was then propagated forward in time to the 5-ms time point using the MSM, which gives the MSM-predicted excited state prior ensemble, **E**_**MSM**_. This ensemble was then refined using EROS against an experimental SAXS profile observed after 5-ms of refolding to obtain our best estimate of the excited state ensemble, **E**_**SAXS**_that incorporates both simulation and experimental information.

### CF and SF-SAXS experiments

CF-SAXS measurements were made as previously described^51^ at the BioCAT beamline at the Advanced Photon Source facility (Argonne, IL), using a compound refractive lens, quartz mixer, and a 12 KeV X-ray source. The total flow rate was 5 mL/min using 1:10 dilution of the unfolded protein to a final protein concentration of 1.8 mg/mL in 10 mM potassium phosphate and 0.8 M urea at pH 7.0. The mixing dead time was 124 μs and the first observable time point was at 172 μs with a time resolution of 42 μs per step and 60–80 frames of 200 ms exposure at each time point. The data were binned over 5 time points to yield the first trace at 255 μs with over 1 minute of exposure.

SF-SAXS experiments used the biological SFM 4000 instrument was used with a 0-0 delay mixer and X-ray head with a 1.1 mm capillary. The energy of the X-ray source was 12 KeV. The experiment was run at a flow rate of 4 mL/sec in continuous flow mode. The exposure time for each frame was 1 second and the experiment was repeated 6 times. The calculated dead time under these conditions was 4 ms. Both CF and SF experiments used the Pilatus 100 detector and the camera length was set to 1.5 M (SF) and 0.5 M (CF).

### Finding the optimal level of EROS refinement from E_MSM_ to E_SAXS_

The EROS algorithm^32^ repeatedly perturbs the microstate weights via simulated annealing to minimize the following objective function, *G*:


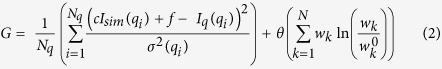


The first term represents the mean-squared error between the simulated and experimental profiles weighted by the experimental error (χ^2^). *N*_*q*_ is the number of data points in the SAXS profile,*cI*_*sim*_(*q*_*i*_) + *f* is the simulated SAXS intensity from CRYSOL at q = q_i,_ where *c* is a normalization constant and *f* is a constant offset to correct for background subtraction^52^. *I*_*q*_(*q*_*i*_) is the experimental SAXS intensity, and *σ*^2^(*q*_*i*_) is the experimental error. The second term is the maximum entropy penalty function that punishes deviations of the weights *w*_*k*_ from the prior ensemble weights 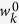. *N* is the number of states in the ensemble, and *θ* is a parameter that determines the strength of the penalty, with a value of *θ* = 0 corresponding to no penalty and best possible fit to experiment, but at highest risk of overfitting. In essence, minimizing *G* corresponds to the process of improving agreement with the experimental data while staying reasonably close to the prior to limit the model’s additional complexity. We employed this regularization penalty due to the fact that default CRYSOL parameters, which were shown to imperfectly predict the CheY* native state (SI Fig. 1), were used to predict SAXS profiles for the simulated structural ensemble. Thus, the best possible fit to experiment in terms of χ^2^ may not necessarily correspond to the ensemble most representative of the true excited state, and may instead be the result of overfitting to the experimental data.

The optimal value of *θ* to estimate the excited state **E**_**SAXS**_ should be an intermediate value that allows for adequate refinement, but avoids significant overfitting. We identified such a value by plotting converged MSE from experiment with respect to *θ* and looking for points of sharp decrease in χ^2^ with decreasing *θ*. This corresponds to the region in which significant deviation from the prior is beginning to occur in favor of improved agreement with the experiment, indicating that the model may be becoming unnecessarily complex (SI Fig. 2, bottom). For the extreme choice of *θ* = 0 (SI Fig. 2, left), it can be seen that the fit is nearly identical to experiment. However, this solution is likely to be overfit due to the error incurred in predicting the individual state profiles using default CRYSOL parameters. Therefore, we chose to compute **E**_**SAXS**_using an intermediate value of *θ* = 6, the smallest value that we deemed could avoid significant overfitting. To test the effects of *θ* on the refinement, we compared the contact maps of the excited states predicted at various values of *θ*. We observe that the contact maps were qualitatively very similar across the entire range of *θ* (SI Fig. 3). This indicates that the key structural features of the excited state were insensitive to the choice of *θ*, and the artifacts introduced by selecting *θ* = 6 are small enough that they do not notably impact the characterization of the excited state. However, we stress that if additional sources of experimental data were available for the system, a better method for selecting the optimal value of *θ* would be to compute the excited state ensemble for each value of *θ* and then cross-validating each ensemble by predicting an independent experimental result. Because this option was not available to us due to scarcity of available experimental data, we chose a reasonable intermediate value of *θ* that balances the levels of refinement and overfitting.

Finally, we must also consider the sensitivity of the refined ensemble to the stochasticity of the EROS refinement algorithm itself. Because the process is a directed random search over all conformational space, it is possible that independent trials of EROS will result in different converged solutions. To investigate whether this stochasticity results in structurally significant differences in our prediction of the excited state ensemble, we ran 20 independent EROS refinements using our designated optimal value of the regularization penalty until convergence, and computed the error between each of the converged **E**_**SAXS**_ profiles. We found that this error was negligible, maximally only 1.2% of the experimental error, and gave resulting ensembles with virtually identical contact maps to **E**_**SAXS**_.

### Simulating FRET experiments

The FRET experiments were predicted using in-house software in combination with the accessible volume (AV) algorithm of Seidel *et al*. that was used to model the accessible position distributions for the dyes after accounting for linker flexibility^53^. The software was benchmarked by computing the native state FRET efficiency histogram of phosphoglycerate kinase (RCSB PDB ID: 3PGK) and comparing to experiment^54^. The parameters used in the simulation (i.e. R_0_, linker length, photons per data point) were set to correspond with those of the experiment. Very good agreement was observed between the simulation and experimental results (SI Fig. 4). This software was then used to predict FRET efficiency histograms for a diverse set of residue pairs for the native, unfolded, and excited states. Donors were placed on K7, S15, K26, E37, G49 and acceptors on M63, S79 K91, S102, K109, for a total of 30 combinations, and R_0_ values ranged between 30 Å and 50 Å. The linker length was set to 15 Å to simulate a standard (CH_2_)_5_ linker. We then selected and analyzed the results from residue pairs and values of R_0_ that could effectively discriminate between the excited state and the native/unfolded states. Of these, we found that dye pairs at K26/M63 with R_0_ = 38 Å, and K7/M63 with R_0_ = 42 Å produced the most informative results. To cross-validate these results experimentally, we recommend using a dye pair such as ATTO-Tec 390/650 (R_0_ = 40 Å) to match our simulation parameters.

## Additional Information

**How to cite this article:** Shi, J. *et al*. Atomistic structural ensemble refinement reveals non-native structure stabilizes a sub-millisecond folding intermediate of CheY. *Sci. Rep.* **7**, 44116; doi: 10.1038/srep44116 (2017).

**Publisher's note:** Springer Nature remains neutral with regard to jurisdictional claims in published maps and institutional affiliations.

## Supplementary Material

Supplementary Information
