# Supplementary material for: Atomistic structural ensemble refinement reveals non-native structure stabilizes a sub-millisecond folding intermediate of CheY
Source: Sci Rep. 2017 Mar 8;7:44116. doi: 10.1038/srep44116 (PMC5341065; doi:10.1038/srep44116)
Supplement: Supplementary Information [file srep44116-s1.doc]

**Atomistic structural ensemble refinement reveals non-native structure stabilizes a sub-millisecond folding** **intermediate of CheY**

Jade Shi, R. Paul Nobrega, Christian Schwantes, Sagar V. Kathuria, Osman Bilsel, C. Robert Matthews, T.J. Lane, and Vijay S. Pande

**Supplementary Information:**

*1) Benchmarking SAXS prediction software*


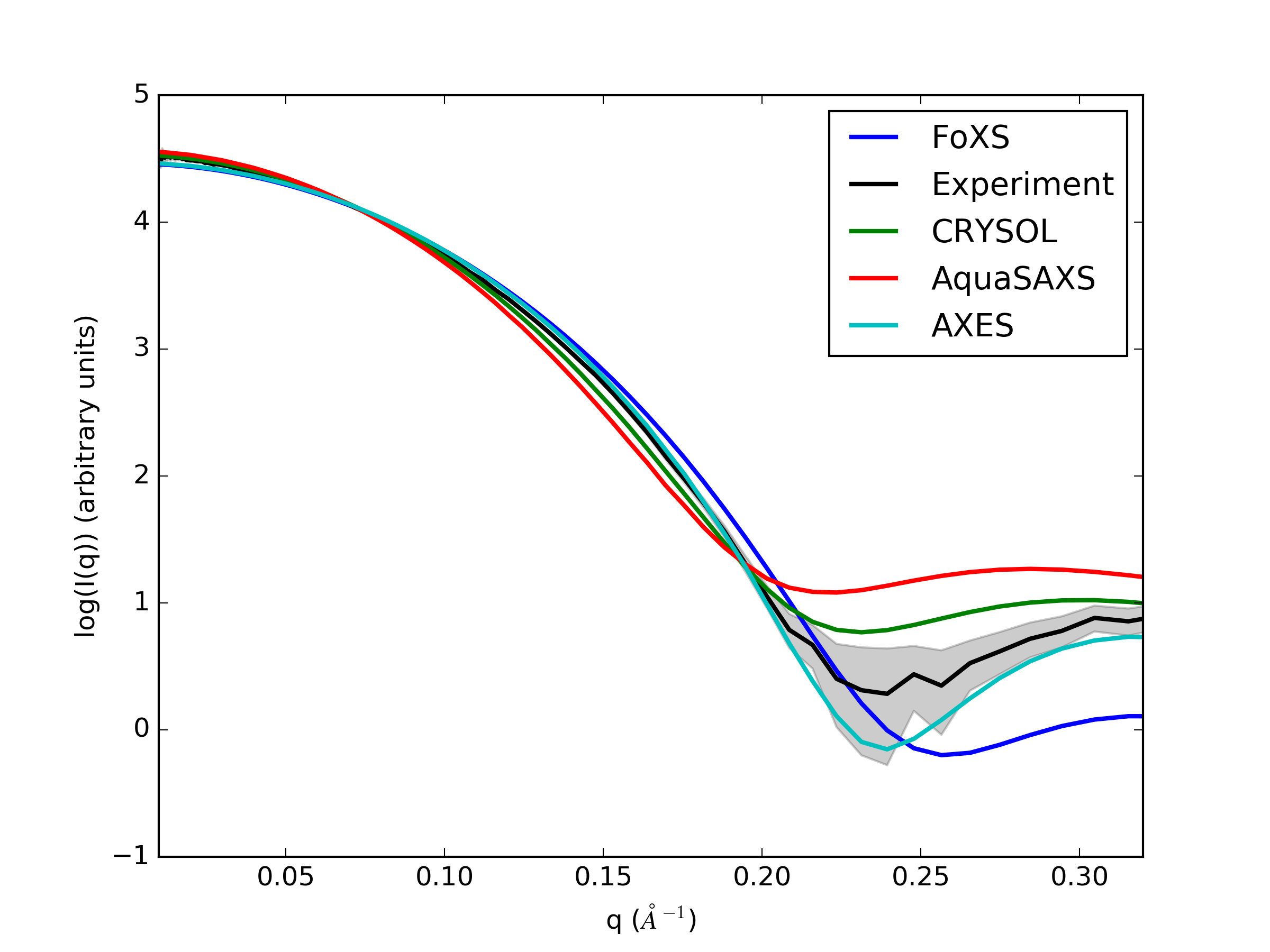


**Figure 1a:** Comparison of four separate SAXS prediction software against the CheY experimental native state profile, using default parameters for all predictors, shows that CRYSOL agrees better with experiment by a considerable margin compared to the other methods. CRYSOL was therefore chosen for this study. The shaded red area represents experimental standard deviation due to repeat measurements.

χ^2^ from experiment:

CRYSOL: **13.4**

AXES: 14.7

FoXS: 29.9

AquaSAXS: 60.6

*
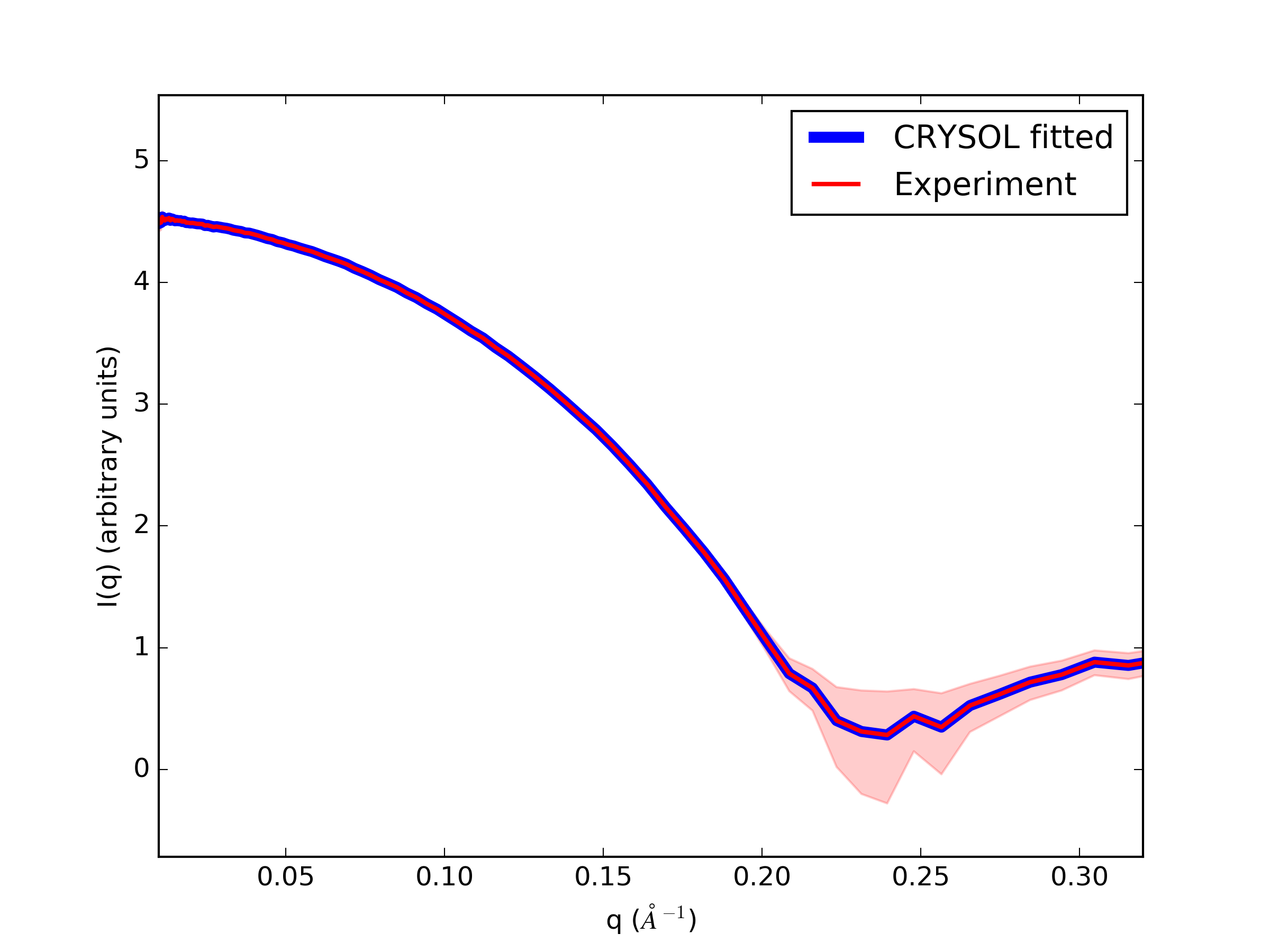
*

**Figure 1b:** Running CRYSOL in fitting mode significantly improves the agreement with experiment (χ^2^ = 1.2). However, these parameters were not used for our study, as they are the result of overfitting to the native state and likely unsuitable for the remaining conformations in our structural ensemble, most of which have very different structures and topologies and require different treatments of solvation and excluded volume. Default parameters were used instead in our study to minimize such potentially unwanted bias. The shaded red area represents experimental standard deviation due to repeat measurements.

*2) Selecting the optimal value of the regularization penalty* θ

*
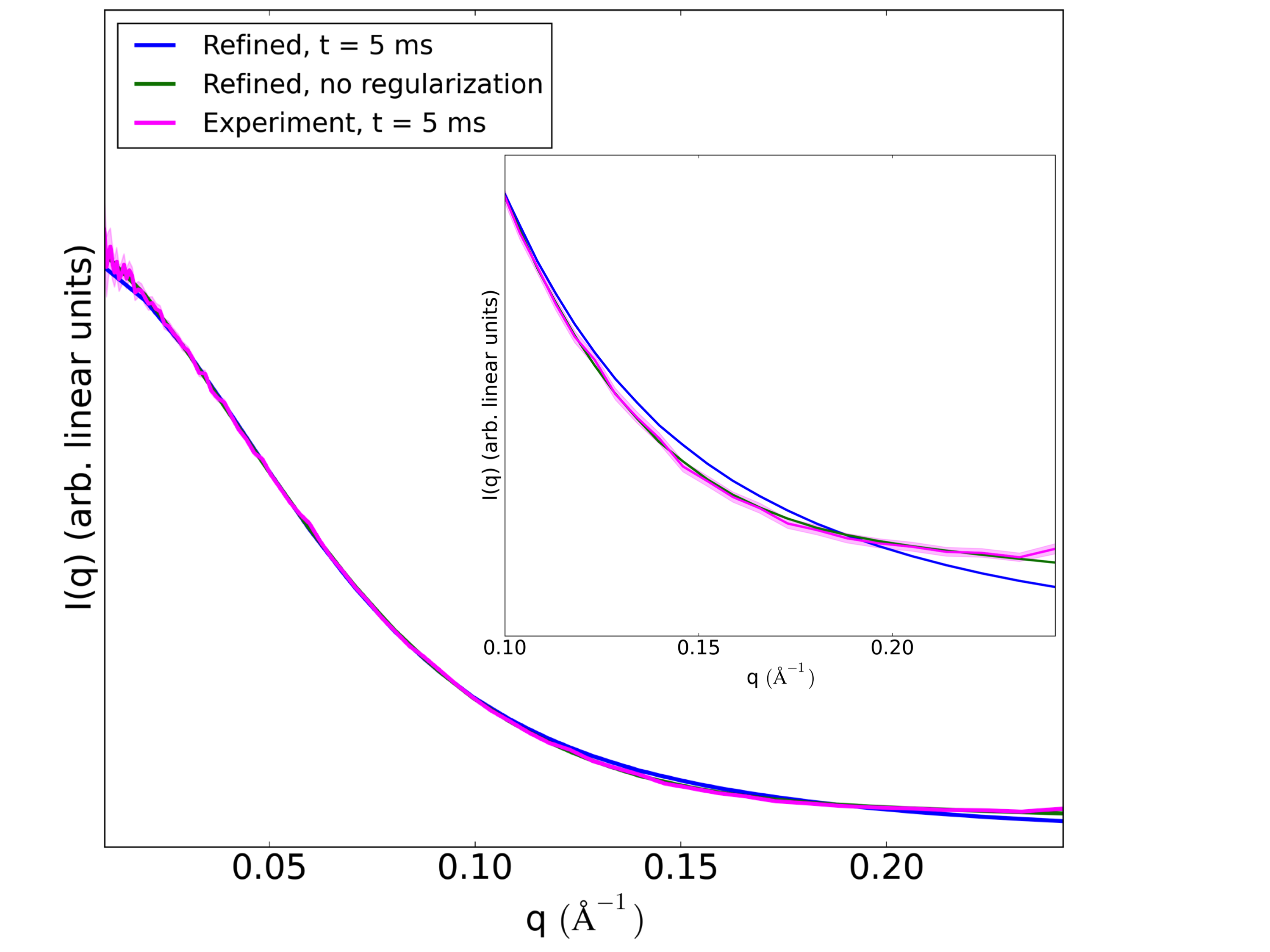
*

**
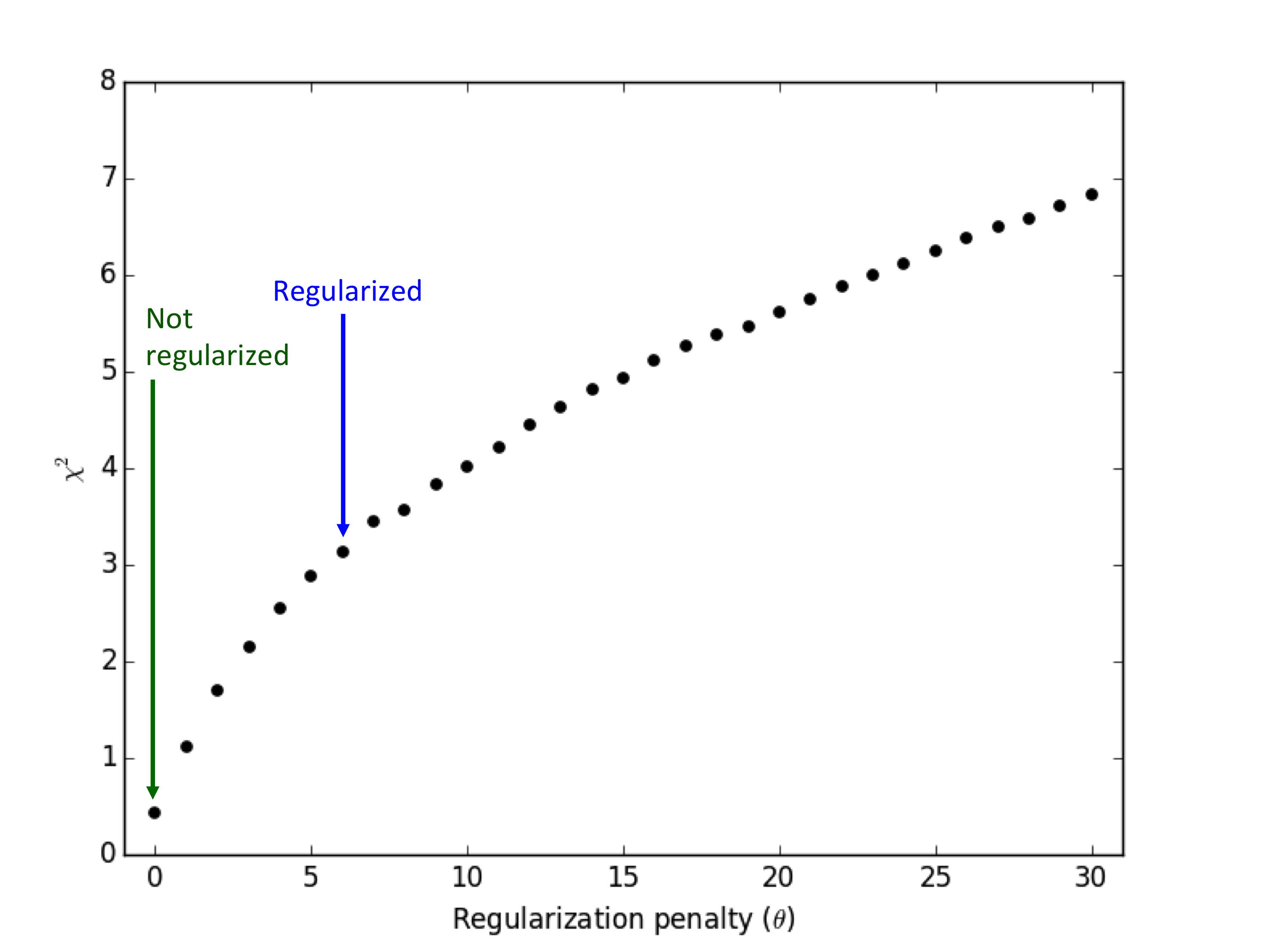
**

**Figure 2:** *Top:* The simulated ensemble SAXS profiles for the unregularized and regularized results are plotted versus experiment. Experimental error is represented as the shaded area around the experimental SAXS profile. With no regularization, the curve is fit much more precisely to experiment (χ^2^ = 0.44) compared to the regularized fit (χ^2^ = 3.13), but this solution is the result of a degree of overfitting, due to the likely inaccuracies made by CRYSOL in predicting the individual SAXS profiles in our ensemble using default parameters. We therefore propose that the regularized solution, which is less consistent with experiment but closer to our informative prior **E_MSM_**, is the preferred solution.

*Bottom* Plots of the χ^2^ error with experiment for different values of the regularization penalty θ. At around θ = 6, χ^2^ begins to sharply increase as θ is decreased, suggesting that this regime with θ < 6 was that in which significant overfitting to experiment was beginning to occur. We therefore picked θ = 6 as the optimal value of regularization to balance consistency with experiment and closeness to the prior.

*3) Sensitivity of E_MSM_ features to* θ

**
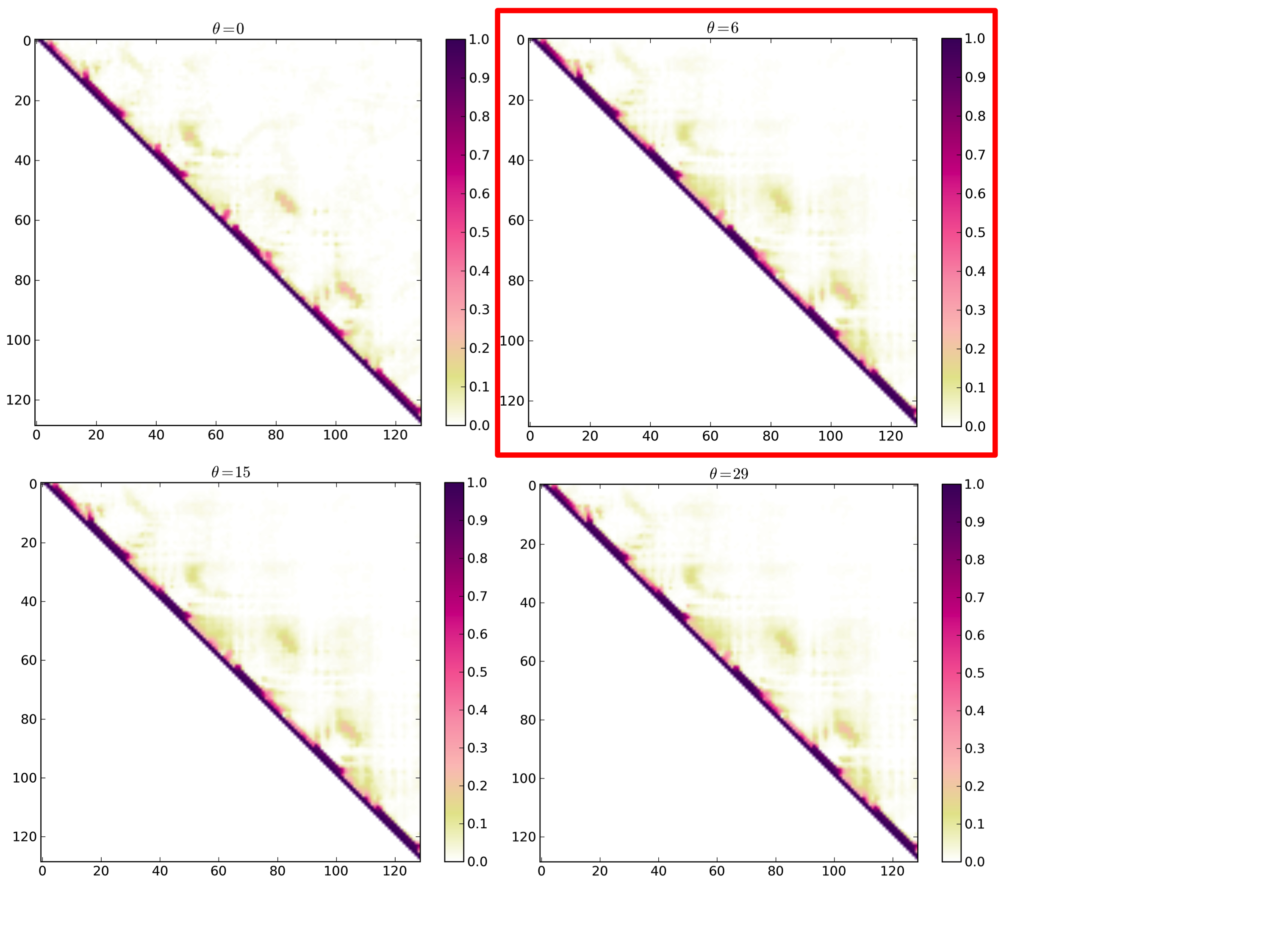
**

**
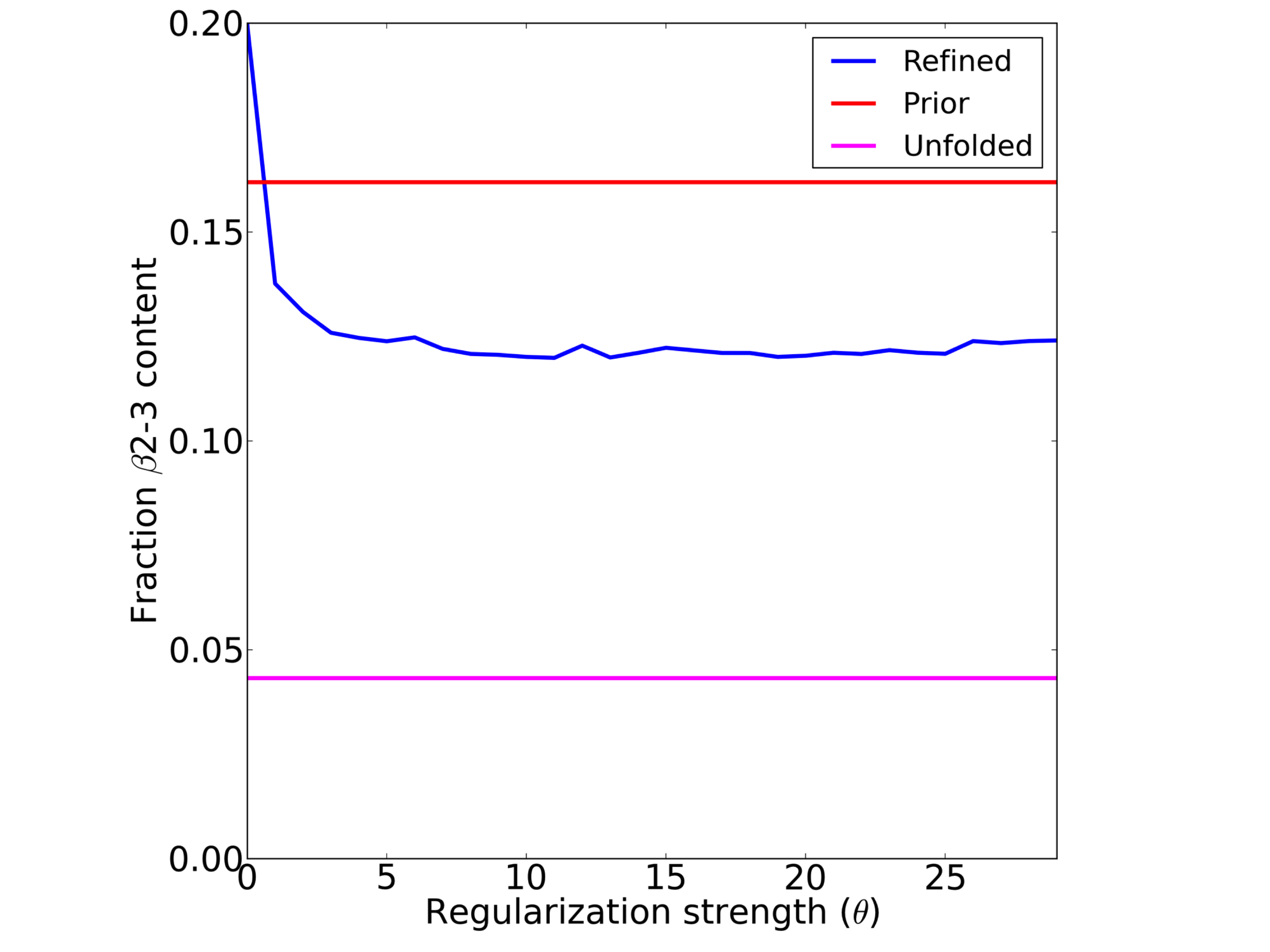
**

**Figure 3:** *Top:* The ensemble contact map of **E_SAXS_** over a range of regularization parameter θ, with our chosen optimal value θ = 6 highlighted in red. The contact maps are only minimally perturbed by the regularization penalty choice and qualitatively contain the same structural features: a marginally formed β3-4 and β4-5 interface, an unstructured N-terminus with minimal β2-1-3 content, and a sizable amount of non-native β2-3 sheet.

*Bottom:* The characteristic non-native β2-3 sheet content remains roughly constant over the range of θ, indicating that the choice of θ only minimally impacts the structural characterization of the excited state. The exception to this is when regularization is completely removed, after which we observe the β2-3 content being upweighted from 12% to 19%. We posit that this is an artifact of overfitting the experimental data.

*4) Benchmarking smFRET prediction software*

**
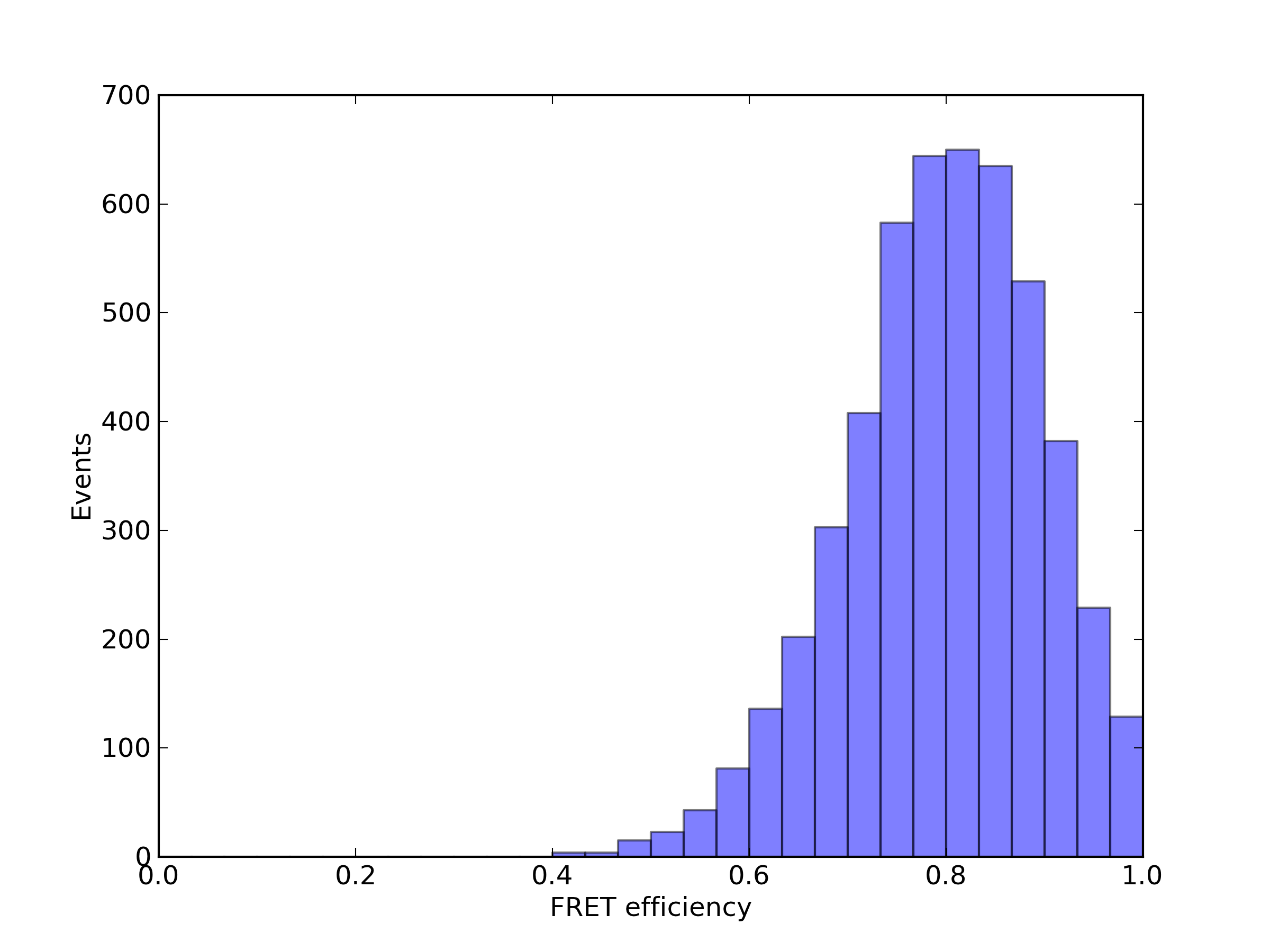
**

**Figure 4:** A simulated single-molecule FRET efficiency histogram for phosphoglycerate kinase (above) was compared to literature results[[1]](#endnote-2) to benchmark the in-house software used to simulate the cross-validation experiments for **E_SAXS_**.

*Simulation parameters used*:

-Donor = Alexa488 (linker length = 8 Å, linker width = 0.5 Å, dye radius = 3.5 Å)

-Acceptor = Alexa647 (linker length = 8 Å, linker width = 0.5 Å, dye radius = 3.5 Å)

-R_0_ = 48.9 Å

-Number of data points taken: 5000

-Photons per burst: 30

*Results*:

μ = **0.78** (experiment = **0.8**)

σ = **0.20** (experiment = **0.23**)

*5) Generating the CheY* unfolded state ensemble*


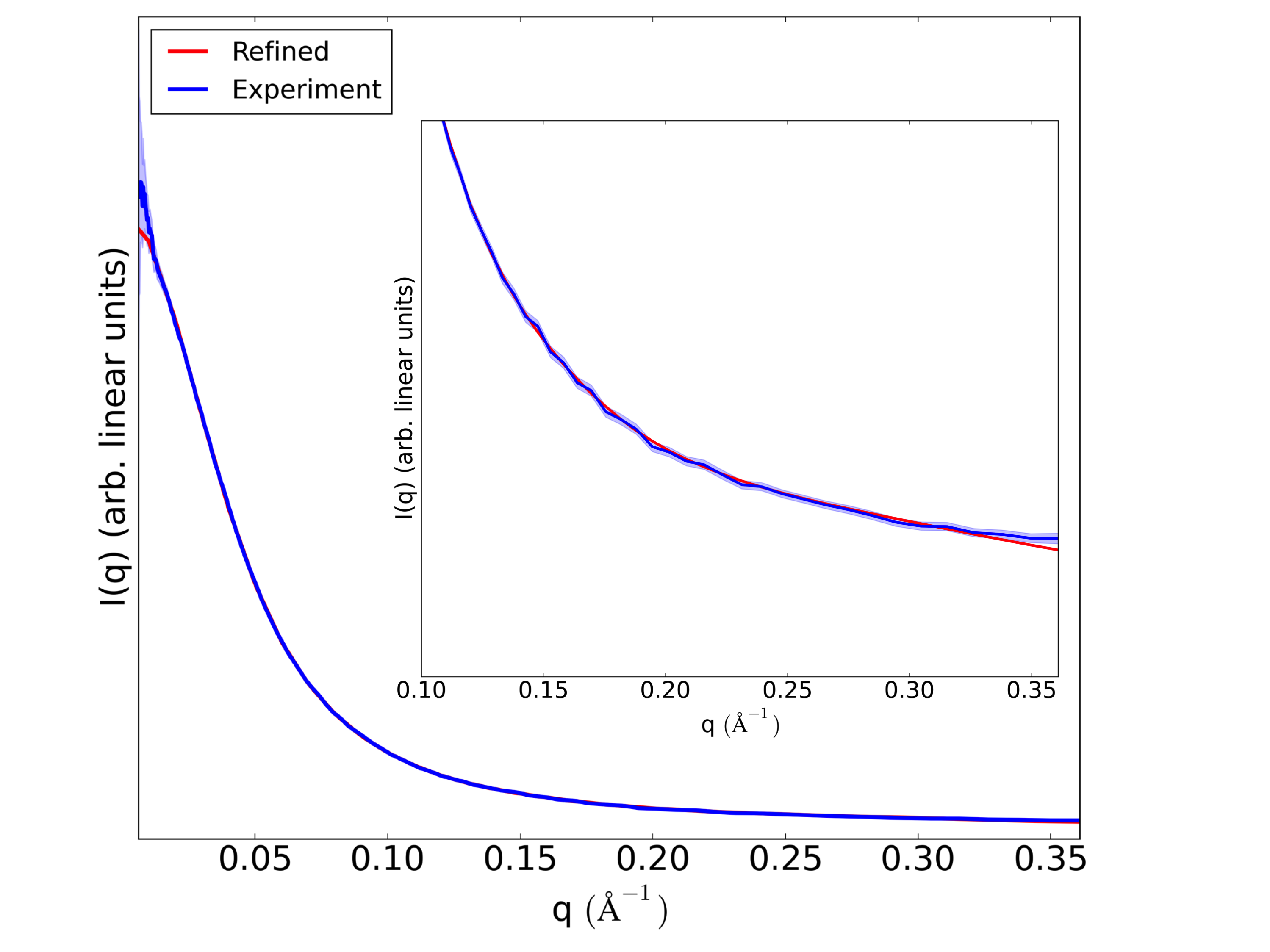


**Figure 5:** The simulated unfolded state ensemble **E_U_** was obtained by refining an arbitrary (uniform-weight) prior to achieve maximum agreement with the experimental CheY* 8M-urea denatured state SAXS profile (χ^2^ = 1.1). Experimental error as a result of repeat measurements is represented as the shaded area around the experimental SAXS profile.

*6) Testing the robustness of excited state features with respect to choice of prior*

*
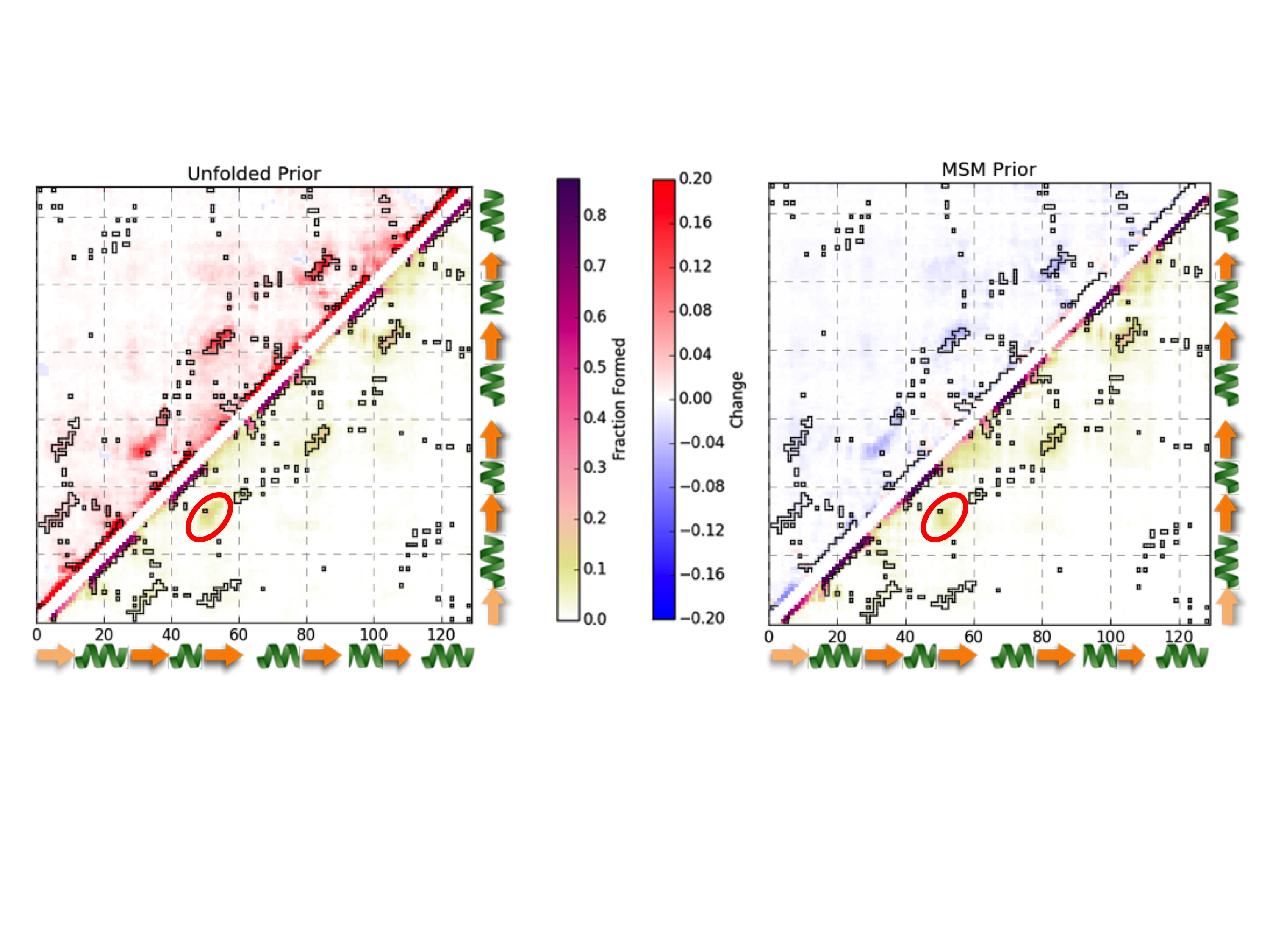
*

**Figure 6:** The robustness of the structural features of **E_SAXS_** to choice of prior was tested by refining two separate priors: the simulated unfolded state **E_U_** (left) and the MSM prior **E_MSM_** (right). Both optimizations qualitatively recover the structural features of **E_SAXS_**. Strikingly, the most characteristic feature of the excited state, the non-native β2-3 sheet (circled in red) that stabilizes the unstructured N-terminus with loose β1/α1 tail is recovered when beginning from an intentionally bad prior, the unfolded ensemble, indicating that this structural feature is robust to choice of initial state populations and is actively up-weighted by the refinement process.

*7) Sensitivity of excited state characterization to default versus fitted CRYSOL parameters*

**
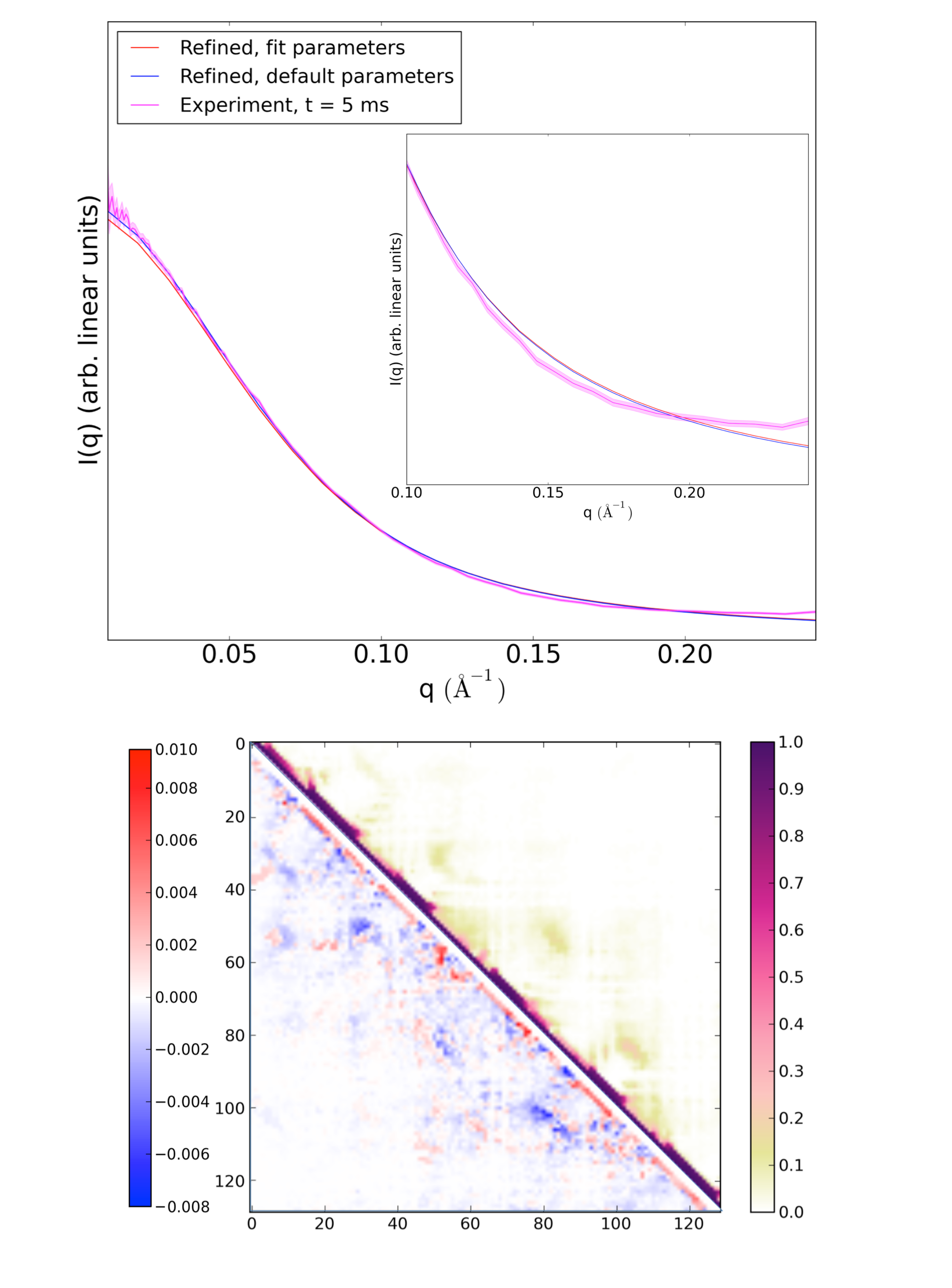
**

**Figure 7:** The SAXS profiles for the structural ensemble using CRYSOL parameters fit to the native state was re-predicted and then a new ensemble SAXS profile for **E_SAXS_** using these profiles and the original refined weights was generated. This results in only minor perturbations to the SAXS profile that are less than the experimental uncertainties due to repeat measurements, represented by the shaded area around the profile (top). Furthermore, re-refining the ensemble using fitted parameters beginning from **E_MSM_** produces a refined ensemble with virtually identical contact map to **E_SAXS_** using default parameters, with contact density per contact varying maximally by 1% (bottom). Thus, our excited state characterization appears to be robust to choice of either default or fitted parameters.

*8) Testing the convergence of the CheY* MD simulation*

**
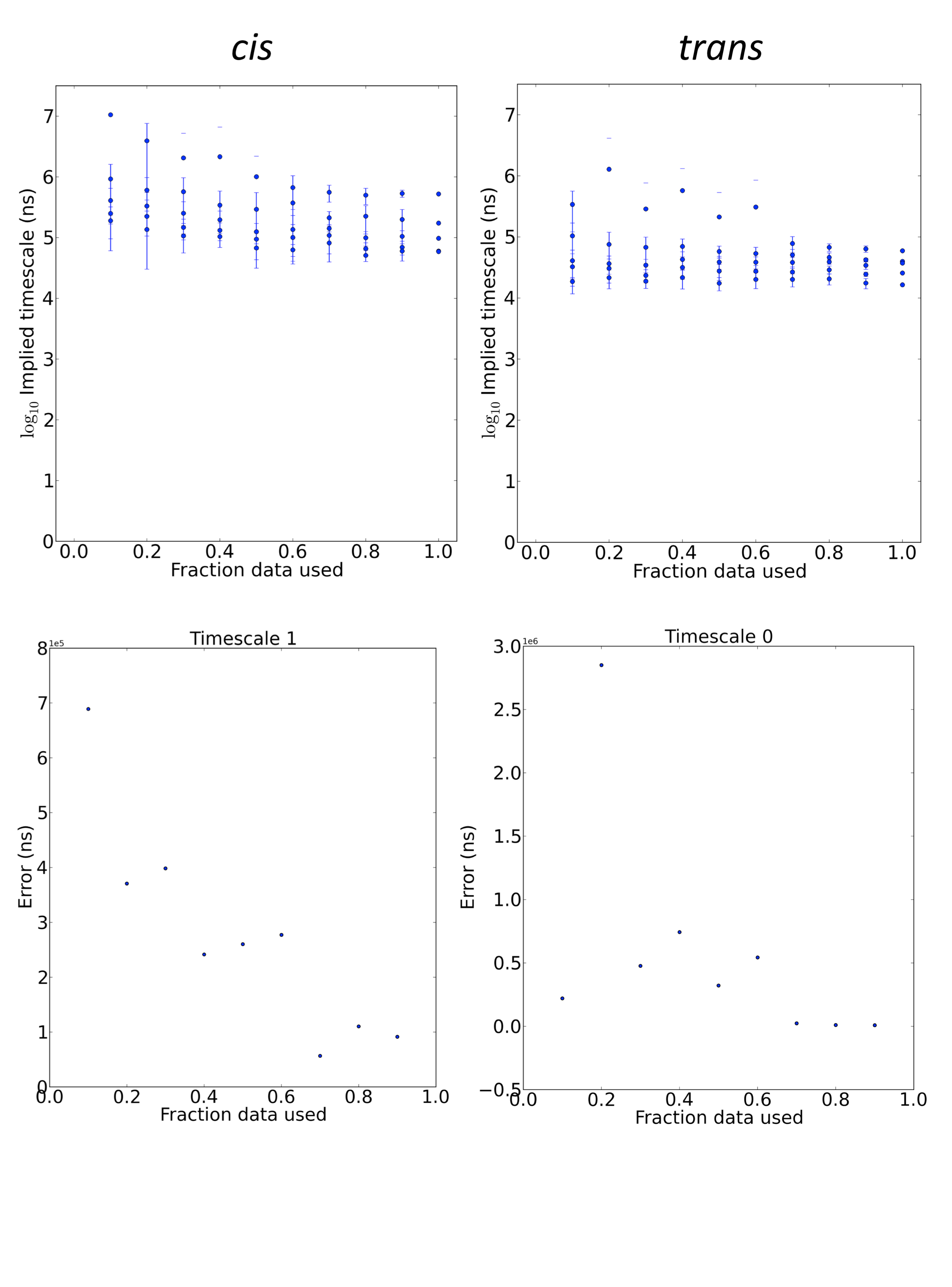
**

**Figure 8:** MSMs were built using subsets of the full CheY* dataset, ranging from 10% to 90% in intervals of 10%. For each percentage value, this process was repeated 10 times using different random samples. The implied timescales for the top five slowest processes predicted by the models (top) and standard deviations for the timescales most closely corresponding to the collapse to the excited state (bottom) are plotted. For both the *cis* and *trans* models, the excited state collapse timescale appears approximately stable if at least 70% of the data is used, suggesting that using at least this amount results in a converged solution with respect to excited state characterization.

**Supplementary References:**

1. Rosenkranz, T., Schlesinger, R., Gabba, M., Fitter, J. Native and Unfolded States of Phosphoglycerate Kinase Studied by Single-Molecule FRET, *ChemPhysChem*, **12**, 704-710 (2011). [↑](#endnote-ref-2)
